# Loop-Mediated Isothermal Amplification (LAMP): An Innovative Approach for the Environmental Monitoring of SARS-CoV-2

**DOI:** 10.3390/pathogens13111022

**Published:** 2024-11-20

**Authors:** Simona Spiteri, Federica Marino, Luna Girolamini, Maria Rosaria Pascale, Carlo Derelitto, Laura Caligaris, Simone Paghera, Sandra Cristino

**Affiliations:** 1 Department of Biological, Geological, and Environmental Sciences, University of Bologna, via San Giacomo 12, 40126 Bologna, Italy; simona.spiteri2@unibo.it (S.S.); federica.marino20@unibo.it (F.M.); luna.girolamini2@unibo.it (L.G.); mariarosaria.pascal2@unibo.it (M.R.P.); carlo.derelitto@unibo.it (C.D.); laura.caligaris2@unibo.it (L.C.); 2 Department of Medical and Surgical Sciences, University of Bologna, 40126 Bologna, Italy; 3 Copan Italia, 25125 Brescia, Italy; simone.paghera@copangroup.com

**Keywords:** SARS-CoV-2, COVID-19, RT-qPCR, RT-LAMP, environmental surveillance, public health

## Abstract

The rapid and accurate detection of SARS-CoV-2 in environmental settings is crucial for effective public health management during the COVID-19 pandemic. This study compares the performance of the Reverse Transcription quantitative polymerase chain reaction (RT-qPCR) and the Reverse Transcription loop-mediated isothermal amplification (RT-LAMP) for SARS-CoV-2 detection from 100 surface samples collected in healthcare environments. The reference method, RT-qPCR, identified a percentage of 25% of positive samples, while RT-LAMP detected a percentage of 27% of positive surfaces. Our findings reveal a sensitivity of 32% and specificity of 75% for RT-LAMP, with a positive predictive value of 30% and a negative predictive value of 77%. The overall accuracy and concordance with RT-qPCR was 64% for both methods. Despite its lower sensitivity compared to RT-qPCR, RT-LAMP had an advantage due to its rapid screening and environmental surveillance, which is particularly useful for confirming negative results. These results underscore the potential of RT-LAMP not only as a valuable method in the environmental monitoring of SARS-CoV-2 but also as a system to control the sanitation process in ordinary and emergency conditions, providing further optimization and validation for its reliability in routine surveillance and outbreak response efforts.

## 1. Introduction

Coronaviruses (CoVs) are a group of viruses known for causing illnesses ranging from the common cold to more severe diseases such as Middle East Respiratory Syndrome (MERS) and Severe Acute Respiratory Syndrome (SARS) [1]. Severe Acute Respiratory Syndrome Coronavirus 2 (SARS-CoV-2) is the strain responsible for the Coronavirus Disease (COVID-19). The COVID-19 pandemic has had a global impact, leading to widespread morbidity and mortality over the last four years. As of June 2024, there have been over 750 million confirmed cases and more than 6.9 million deaths worldwide [2]. The rapid spread of the virus prompted unprecedented public health measures, including lockdowns, social distancing, and the widespread use of face masks [3]. Despite these efforts, the virus’s high transmission rate and asymptomatic spread posed significant challenges when it came to controlling the pandemic. The disease manifests in flu-like symptoms such as fever, cough, dyspnea, and gastrointestinal disorders, which can evolve into systemic inflammation and multiorgan dysfunction. The history of COVID-19 begins in late 2019 in Wuhan, China [4]. On 31 December 2019, Chinese health authorities announced a pneumonia outbreak of unknown etiology traceable to the seafood and wildlife city market, where mammalian species were on sale. This circumstance suggested a spillover from animals to humans. For example, although the origin of SARS-CoV-2 is still under investigation, the clear similarity (96%) between SARS-CoV-2 and two SARS-like CoVs found in bats (bat-SLCoVZX45 and bat-SL-CoVZX2) indicate that spillover events in zoonotic CoVs may have occurred [5]. SARS-CoV-2 is highly transmissible and primarily spreads through respiratory droplets, even if other routes of transmission, including surfaces, have been studied. For example, CoVs are also known for their gastrointestinal tropism. More recently, the presence of SARS-CoV-2 RNA has been found in human feces, suggesting a possible oro-fecal transmission. The main transmission route of human CoVs involves respiratory droplets: patients present a cough, then lung-ground glass opacities, and finally, a progression to severe pneumonia. This is because SARS-CoV-2 replicates in both the upper and lower respiratory tract. In this way, an infected patient can spread contaminated droplets coughing and facilitate the human-to-human transmission of COVID-19. Transmission via direct contact with contaminated surfaces was also reported during the SARS epidemic. Virus-contaminated aerosols adhere to surfaces, involving fomites in the transmission of the pathogen [4]. Currently, there are different opinions on the role of fomites in the transmission of SARS-CoV-2: some authors have demonstrated a minimal risk of contamination, while other authors suggest a higher risk [6]. It has also been proven that SARS-CoV-2 persists on inanimate surfaces like other nosocomial pathogens: hydrophobic and non-porous surfaces (e.g., plastic, metal, glass, ceramics, and rubber surfaces) keep it in stable conditions at room temperature preserving its infectivity for several days depending on the type of surface involved (e.g., on metal or plastic surfaces it can persist for up to 3–4 days) [6,7,8,9,10,11,12]. Environmental surveillance of SARS-CoV-2 has emerged as a critical component in understanding the viral transmission dynamics and preventing future outbreaks. Monitoring the virus’s presence in the environment, such as in air, wastewater, and on surfaces, provides valuable information on potential exposure risks and the effectiveness of sanitation measures. At present, most environmental monitoring efforts are focused on Wastewater-Based Epidemiology (WBE). Wastewater surveillance involves the analysis of sewage samples for the presence of SARS-CoV-2 RNA, which can be shed in the feces of infected individuals, both symptomatic and asymptomatic. WBE permits the detection of a viral load increase in a community before clinical cases are reported. This is particularly valuable for identifying emerging outbreaks and implementing control measures in a timely manner [13,14].

Several studies demonstrated the role of air quality monitoring in viral particles spread in indoor environments, with the aim of mitigating the risk of airborne transmission. These results provide the importance of public health interventions, such as improving ventilation in schools, offices, and public transport systems, or implementing targeted lockdowns in high-risk areas [15,16,17].

The persistence of SARS-CoV-2 on surfaces has been a topic of significant research interest. Environmental monitoring has shown that the virus can survive on various surfaces, such as plastic, stainless steel, and cardboard, for several hours to days under certain conditions. This has important implications for the disinfection protocols in public spaces, workplaces, and homes. By understanding which surfaces are most likely to harbor the virus and under what conditions, public health authorities can develop targeted cleaning and disinfection guidelines to reduce the risk of fomite transmission [6,7].

Among these, surface sampling is particularly important considering that they can support the indirect transmission of the virus. Considering this, surface sampling could be used to understand the SARS-CoV-2 presence in community environments such as healthcare facilities (HCF), long-term care facilities (LTCF), schools, work environments, and leisure centers, to estimate the risk of transmission and trace the chain of infection. Moreover, assessing the presence of the pathogen on surfaces could contribute to evaluating the effectiveness of cleaning protocols. However, one of the main challenges in environmental surveillance is the lack of detailed guidelines on the methods to be used. If several local and international guidelines for the clinical management of COVID-19 are available [18,19,20,21], the same cannot be said for the environmental monitoring of SARS-CoV-2. Despite the magnitude of past events, no definite guidance is available yet to evaluate the spread of SARS-CoV-2 in the environment and to assess the associated risks. Once the transmission routes have been ascertained, environmental monitoring could be carried out by studying the contamination of wastewater, surfaces, and air. In April 2022, a World Health Organization (WHO) Interim Guidance was published. This document, entitled “Environmental surveillance for SARS-CoV-2 to complement public health surveillance” [22], declares the absence of a universal standard for environmental monitoring of SARS-CoV-2 even though national programs and protocols are also available such as the Centre for Disease Control and Prevention (CDC). Unfortunately, the WHO guidance is limited to wastewater surveillance and its purpose [22]. The WHO also provided operating instructions for the study of surfaces as early as February 2020, through the document entitled “Surface sampling of coronavirus disease (COVID-19): A practical “how to” protocol for health care and public health professionals”. This guidance, in addition to sampling sites and the size of the sampling area, a surface of 25 cm^2^, also suggests using a swab with a synthetic tip and a plastic shaft, as well as a neutralizing buffer, to counteract the effects of any residual disinfectant (e.g., Tween 80). If for sample collection the WHO guidance previously described is available, no further guidance is established for sample analysis [23]. In several studies, the most commonly used techniques for detecting SARS-CoV-2 belong to the field of molecular biology. Many studies refer to the use of the Reverse Transcription quantitative polymerase chain reaction (RT-qPCR), relying on the amplification of a predetermined target [24,25,26]. Among the most commonly targeted genes at the beginning of the pandemic were the RdRP and E genes. Following the publication of the first SARS-CoV-2 sequences in the GISAD database in January 2020, the Pasteur Institute published a procedure for the (clinical) detection of SARS-CoV-2, indicating the primer and probe sequences used for the RdRp and E genes of SARS-CoV-2 [27]. Until recently, the S gene was widely used before the “Spike gene target failure (SGTF)” discovery. The SGTF is described in a European Centre for Disease Prevention and Control (ECDC) and WHO update entitled “Methods for the detection and characterization of SARS-CoV-2 variants–first update”. Some of the SARS-CoV-2 VOCs (i.e., Alpha [B.1.1.7] and Omicron [B.1.1.529]) generate a negative or significantly weaker positive S-gene result in multiplex RT-qPCR assays, with positive results for the other targets [28]. This has been used as an indicator or screening method to identify these variants. On the one hand, this would represent a distinguishing factor for certain VOCs, but on the other hand, it can lead to false negative results. The best option is represented by the N gene: it is well known that the N gene represents a well-conserved portion of the SARS-CoV-2 genome. Grifoni et al. (2020) demonstrated that the N gene is one of the most conserved regions of the SARS-CoV-2 genome and, as a consequence, it is suitable for pathogen detection [29]. For further details, please refer to Song et al. (2023) [30].

Thus, while RT-qPCR remains the gold standard for the diagnosis of SARS-CoV-2, there is still no referred standard for the detection of SARS-CoV-2 in environmental settings and on surfaces in particular. If RT-qPCR is a sensitive and specific method that ensures high diagnostic accuracy, making it the preferred method for COVID-19 diagnosis [31], applying RT-qPCR to environmental samples poses several challenges. Environmental samples often contain inhibitors like phenolic compounds, heavy metals, and humic acids that can affect the efficiency of the reaction, and the concentration of viral RNA on surfaces may result lower than in clinical specimens [32]. Moreover, during the early stages of the pandemic, RT-qPCR kits specifically designed for surface sampling were not readily available, forcing the use of clinical diagnostic kits. Given the limitations of RT-qPCR for environmental sampling, alternative methods, such as Reverse Transcription loop-mediated isothermal amplification (RT-LAMP), have gained attention. RT-LAMP is a rapid and cost-effective technique. It provides the nucleic acids with amplification at an isothermal temperature, eliminating the need for thermal cycling. This method is highly specific and can produce results within 30 to 60 min, making RT-LAMP a faster method than RT-qPCR and conventional PCR [33]. During the RT-LAMP analysis, the amplification and detection of the nucleic acid are carried out in a single step using four or six specifically designed primers and a polymerase with strand displacement activities. As opposed to LAMP for DNA, RT-LAMP requires a reverse transcriptase to convert RNA into a complementary DNA (cDNA) used for amplification. Due to this process, massive amplification is possible, as DNA can be amplified up to 10^9^ times in one hour [34,35]. Moreover, RT-LAMP’s robustness against inhibitors present in environmental samples enhances its applicability for surface sampling [36]. Despite the various advantages that RT-LAMP offers, the technique has been widely used for SARS-CoV-2 diagnosis [37,38,39,40] but is not yet common for environmental monitoring. In 2021, a group of South American researchers conducted a study on the environmental monitoring of SARS-CoV-2 in public areas and transportation in Ecuador using RT-LAMP, targeting the nucleocapsid (N2) and envelope (E1) genes. In total, 28 out of 300 (9.33%) surfaces tested positive for SARS-CoV-2, highlighting the importance of environmental monitoring in communal settings [41].

Considering this, the comparison of these molecular techniques aims to assess the performance of innovative detection methods and understand their strengths and limitations for routine environmental monitoring of SARS-CoV-2. This study also aims to clarify the potential use of rapid and innovative technologies as a routine environmental surveillance system, not only for epidemiological purposes but also to assess the hygienic conditions of community settings for comprehensive transmission risk management. Assessing the environmental presence of SARS-CoV-2 allows us to raise awareness among institutions such as the WHO, the Centers for Disease Control and Prevention (CDC), and national health agencies about the importance of developing clear and detailed guidelines for monitoring this and other microorganisms that could potentially cause future outbreaks or pandemics. Finally, this study aims to urge manufacturers to focus their research and development efforts on environmental monitoring of viruses and bacteria, as the detection of microorganisms in the environment is the key to understanding and preventing human infections.

## 2. Materials and Methods

### 2.1. Sampling Method

One hundred surface samples were collected in the city of Bologna, Italy, from HCF and LTCF in the presence of patients hospitalized for complications of COVID-19 or in isolation after a nosocomial COVID-19 infection. The sample collection phase was conducted between January 2022 and March 2023. According to the WHO Guideline for the surface sampling of the virus [22] on regular surfaces, samples were collected using 25 cm^2^ templates, while for small objects or irregular surfaces, the entire surface was sampled. To obtain negative and positive surface control, a surface treated by steam autoclave and positive swab samples were used. In contrast to the WHO guideline, a different type of swab was used, the FLOQSwabs^®^ flocked swab (COPAN, Brescia, Italy) instead of the classical rayon swab. Once the swab was collected, it was stored and transported in a sterile tube following the WHO guidelines, using 3 mL of transport medium. In this study, an SRK viral transport medium (COPAN) was used. After the sample collection, the swabs were transported at 4 °C to the BSL-2 laboratory, for the sample inactivation in a thermal bath at 50 ± 5 °C for 30 min and stored at −80 °C until use [42].

### 2.2. RT-qPCR: SARS-CoV-2 RNA Extraction and Analysis

In order to carry out RT-qPCR, SARS-CoV-2 RNA was first isolated from 140 μL of the SRK medium using the column-based QIAmp Viral Mini kit (Qiagen, Hilden, Germany) according to the protocol for the purification of plasma, serum, urine, cell culture media, or cell-free body fluids using a microcentrifuge. For the extraction process, QIAcube (QIAGEN) was used, the automated nucleic acid system compatible with the QIAmp Viral Mini kit. The recovered RNA (60 µL) was stored at −80 °C until analysis. Given the absence of specific kits for pathogen detection on surfaces at the time the study began, the Allplex SARS-CoV-2/Flu A/Flu B/RSV Assay (Seegene, Seoul, Republic of Korea) was used for RT-qPCR analysis. The Allplex RT-qPCR kit is validated for in vitro diagnostic use to detect *N*, *RdRP*, and *S* genes for SARS-CoV-2, and, at the same time, the presence of other viruses such as influenza A, influenza B, and respiratory syncytial virus (RSV) with a limit of detection (LOD) of 0.23 TCID50/mL for each target. The total volume of the reaction was 20 µL, containing 10 µL of RNA template. For each analytical session, reaction controls (positive and negative) were processed simultaneously with the surface samples. In particular, three negative controls were processed: the Negative Control (NC) provided by Seegene assay; the No Template Control (NTC) as a contamination control, prepared using nuclease-free water added to the PCR Master Mix; and RNA extracted by a swab sample placed on a sterilized surface. Moreover, in each session, two Positive Controls (PCs) were tested. The Seegene assay provides a PC constructed using plasmids encoding Allplex™ SARS-CoV-2/FluA/FluB/RSV Assay target sequences. Since the PC included in this assay is a high concentration PCR control, before each single run the PC is diluted with sterile TE buffer (not provided by the manufacturer) by 1:100. The second PC consists of a positive human swab sample, obtained by one of the authors under personal consent. To confirm the validity of each PCR run on the same plate the user must confirm the results of the negative and positive controls through the Seegene Viewer Software (Seegene, Software version V3.28.000.011), the manufacturer’s software for the RT-qPCR analysis. If the positive and/or negative control results are invalid, the corresponding PCR run was repeated. Reaction conditions were as follows: 50 °C for 20 min, then 95 °C for 15 min followed by 3 cycles of initial PCR activation at 95 °C for 10 s, 60 °C for 40 s, 72 °C for 20 s followed by 42 amplification cycles of 95 °C for 10 s, 60 °C for 15 s, and 72 °C for 10 s. The results of the analysis were interpreted as positive if one or more genes were detected with a cycle threshold (Ct) value ≤ 40. The data regarding the primers and other technique parameters were under intellectual propriety rights by Seegene.

### 2.3. RT-LAMP

RT-LAMP analysis was performed using the environmental SARS-CoV-2 kit (Enbiotech Group S.r.l., Palermo, Italy) with ICGENE PLUS portable platform (Enbiotech Group), consisting of a real-time fluorimeter, monitored and regulated by the ICGENE application (Enbiotech Group, IC-Gene app version 3.5.6-indium) downloadable on various smart devices. The assay was validated for surface use at the Environmental and Health Service Laboratory by the local agency, Agenzia Regionale per la Protezione dell’Ambiente (ARPA), of the Lazio region in Italy. The targets were the *N* and *S* genes and have a limit of detection of 2 copies/μL. The kit package contains the following reagents: PCR tubes, a vial of extraction buffer, a vial of nuclease-free water, a vial of Primer Mix, a vial of mineral oil, and two reaction controls (positive and negative). Upon first use of the kit, only the positive control requires resuspension in 130 µL of nuclease-free water, while the negative control is ready to use. The data regarding the primers and other technique parameters were under the intellectual propriety rights of the Enbiotech Group.

Sample preparation involves extracting viral RNA by soaking the swab tip into a vial containing 200 µL of extraction buffer, which has been labeled with the sample ID. The swab is then discarded, and the vial with the extraction buffer is incubated for 10 min at room temperature. After RNA extraction, 20 µL of the RNA extract is added to previously prepared Primer Mix tubes containing 20 µL of LAMP Mix and 30 µL of mineral oil. The mineral oil helps prevent evaporation and the dispersion of sample droplets during amplification, ensuring fluorescence detection during the analysis. In each session, two reaction controls—a negative control and a positive control—are processed along with the samples, as if they were separate samples: 20 µL of the reaction control is added to Primer Mix PCR tubes containing 20 µL of LAMP Mix and 30 µL of mineral oil. A positive RT-LAMP result is indicated by a sigmoid curve for each sample on a tablet equipped with the IC-Gene app, as well as a “+” sign on the screen.

### 2.4. Statistical Analysis

RT-qPCR and RT-LAMP results were analyzed in terms of positive and negative samples for the comparison of the two techniques; RT-qPCR, the clinical gold standard, was used as the reference method and descriptive statistical analysis was performed. The percentage of positive and negative samples for each technique, the overall concordance (OC), the concordance using the standardized Cohen’s coefficient (k) to measure the level of real agreement between two qualitative measurements [43], the sensitivity (SE) and specificity (SP) and the respective 95% confidence interval (CI), the positive (PPV) and negative predictive values (NPV), were calculated [44].

## 3. Results

After RNA extraction, each sample was analyzed with RT-qPCR and RT-LAMP. Each method returned a qualitative result (positive or negative). For the RT-qPCR technique, 25/100 (25%) samples tested positive, while 75 (75%) tested negative. By contrast, the RT-LAMP technique identified 27/100 (27%) positive samples and 73 (73%) negative samples. This preliminary comparison showed how RT-qPCR detected a similar number of positive samples with respect to RT-LAMP (25 versus 27). In order to assess the performance of RT-LAMP with respect to RT-qPCR, the overall concordance (OC) (Table 1) and Cohen’s Kappa (k) measure of agreement among techniques (Table 2) were calculated using a contingency table.

The percentage of concordance results showed a higher number of concordant negative samples (true negatives, TN) than the positive concordant samples (True positives, TP). An agreement percentage of 64% is observed for the two methods under investigation. This value is confirmed by Cohen’s k of 0.0649 which corresponds to the slight agreement of Cohen’s k interpretation scale.

Using the contingency table previously reported (Table 2), the performance of RT-LAMP with SE, SP, PPV, NPV, and ACC were calculated, as reported in Table 3 [44].

Comparing the two methods, RT-LAMP showed a SE of 32.0% and a SP of 74.7%. These values suggest that RT-LAMP was generally effective in detecting TN samples. In line with the SE and SP values obtained, the PPV and NPV, respectively, at 29.6% and 76.7% suggest that negative results from RT-LAMP are highly reliable. A total of 64% of the ACC and the OC of RT-LAMP suggest that the method provides correct results in most cases, contributing on average to the ability to correctly identify the presence or absence of the viral target, while Cohen’s k of 0.06 indicates a fair level of agreement between the two methods.

## 4. Discussion

SARS-CoV-2 has significantly impacted global public health, economies, and daily life. Understanding the transmission dynamics of SARS-CoV-2, particularly in community environments, is crucial for controlling outbreaks and preventing further spread. Environmental monitoring of the virus on surfaces represents an important public health strategy, as it helps identify potential outbreaks and implement sanitization protocols to mitigate transmission risks [25].

The comparison between RT-qPCR and RT-LAMP techniques for detecting SARS-CoV-2 on surface swab samples from community environments revealed important insights into the performance of these methods. A total of 100 surfaces samples were collected and analyzed with two different techniques: the clinical gold standard RT-qPCR and the rapid and innovative RT-LAMP. The RT-qPCR analysis, considered the reference method for the comparison with RT-LAMP, identified 25 positive and 75 negative samples. In contrast, RT-LAMP detected 27 positive and 73 negative samples.

This comparison between RT-qPCR and RT-LAMP shows a similar number of negative samples between the techniques. Although the number of positive samples for the compared techniques is similar with an OC of 64%, the measure of rater reliability shows a slight agreement. However, Cohen’s K has some limitations, considering that sometimes a study reporting a high percentage of concordance can present a low kappa value, proving inconsistent. The reason for this statistical phenomenon is the first kappa paradox, caused by the effect that the prevalence of the subject under study has in a dataset. Because of this feature, an imbalance in case distribution will render lower kappa values. Moreover, in molecular biology, certain errors may occur because of specific characteristics of the methods or samples analyzed. Additionally, the kappa value can be influenced by sample size: smaller samples may result in a less reliable and more variable K value. Cohen’s K limitations underscore the importance of using complementary methods for a more robust and reliable evaluation [45,46,47,48,49]. For this reason, SE, SP, PPV, NPV, and ACC were assessed. The comparison shows an SE percentage of 30% and an SP of 75%. Despite the low percentage of SE, the higher percentage of SP suggests that RT-LAMP could be reliably used to trace truly negative surfaces. In fact, RT-LAMP is considered a highly specific technique due to the use of a larger number of primers than classical PCR. Having more than two primers permits the recognition of a higher number of target RNA regions, making the amplification reaction more specific [50]. These considerations about SP are reflected in the NPV of RT-LAMP: the obtained NPV indicates that 77% of the samples that test negative with RT-LAMP are truly negative according to RT-qPCR. This value indicates a greater reliability of RT-LAMP when it detects a negative result. The OC and ACC, both at 64%, suggest a fair concordance and a moderate overall performance.

These results should be evaluated in light of the differences between the compared techniques and COVID-19 prevalence. Regarding the differences among both techniques, the RT-LAMP and RT-qPCR analysis were carried out using two kits detecting different targets. For RT-qPCR, the Allplex SARS-CoV-2/Flu A/Flu B/RSV Assay (Seegene) was used. It is a clinical multiplex RT- PCR assay designed to detect N gene, RdRP gene and S genes for SARS-CoV-2, influenza A, influenza B, and respiratory syncytial virus (RSV). For RT-LAMP, the environmental SARS-CoV-2 kit (Enbiotech) was used to target SARS-CoV-2 N and S genes. During the development of SARS-CoV-2 detection kits, the N target is often used because it represents a highly conserved portion of the genome [29]. Both kits used in this study detect N targets alongside the *RdRp* and *S* genes. Nevertheless, targeting the S gene can pose a challenge for pathogen detection due to the issue of Spike Gene Target Failure (SGTF) [28]. In the early years of the pandemic, as the clinical SARS-CoV-2 kits were developed and licensed for Emergency Use by the Food and Drug Administration (FDA) (such as the RT-qPCR kit used in this study), the SGTF phenomenon emerged. Moreover, the sampling campaign was conducted in HCFs and LTCFs where, due to local authority regulations, consistent access was not possible. This led to sampling at different points during the pandemic, with variable disease prevalence rates. This, combined with the rapid mutation of SARS-CoV-2, may have contributed more to the failure of some VOC detections than those covered by the kit validation. As a consequence, the detection failure of TP samples by both techniques results in a decrease not only in Cohen’s Kappa value but also in SE, SP, PPV, NPV, and ACC.

In addition to these challenges, a potential matrix effect should be considered: surfaces often contain substances that can interfere with amplification processes [32]. This may have occurred during the present study, as the RT-qPCR assay is designed for clinical use.

Despite the limitations of this study, these results demonstrate that a synergy between clinical and environmental approaches permits them to overcome the challenges of the emerging disease overall monitoring.

RT-LAMP analysis represents a handy, rapid, and cost-effective technique. It requires at least three primer pairs per target, conferring greater specificity to the assay. This would make RT-LAMP a very advantageous technique in several respects. Unlike RT-qPCR, RT-LAMP requires an isothermal amplification process at a temperature of about 65 °C. The three main steps that characterize classical amplification reactions (melting, annealing, and elongation). This, together with the use of specific buffers for RNA extraction, would greatly reduce the analysis and response time from more than 6 h to about one hour [35]. In addition, because there are no different amplification cycles with different temperatures, LAMP technology does not require the use of sophisticated instrumentation such as a thermocycler, optics for fluorescence excitation and emission collection, and data acquisition and analysis software; a thermostat bath or thermal block at a constant temperature would suffice. Since sample preparation and analysis do not require an experienced operator, the IC-GENE system, employed in this study, merges the advantages of LAMP technology with user-friendliness, making it accessible for every operator. The portability of the IC-GENE platform allows on-site testing, meaning that analyses can be conducted at the sampling location. This results in faster analytical execution and a reduction in turnaround time (TAT). Optimizing TAT directly translates to a more efficient workflow post-LAMP analysis. For instance, in diagnostics, a shorter TAT enables faster access to appropriate antibiotic therapy for patients. Environmental monitoring, such as testing air and surface samples, helps quickly detect nosocomial pathogens in hospital environments, enabling the prompt implementation of sanitization measures. However, despite the numerous advantages offered by the technology examined, the results show a moderate specificity value (75%) and a low sensitivity value (32%). These values must be considered in relation to the type of technique used, the target examined, and the applications of LAMP. In fact, LAMP is a method designed and used when the evaluator prioritizes specificity over sensitivity. In the case of environmental monitoring for SARS-CoV-2, the virus is outside of its host cell, with an estimated half-life of several days (depending on the surface properties) outside the host [7], lacking the resources necessary for replication and being subject to the degrading effects of physical and mechanical agents [51].

In this context, detecting SARS-CoV-2 RNA in the environment allows us to promptly identify potentially hazardous contamination, allowing for timely sanitation procedures in high-risk areas. Although the WHO has declared the end of the COVID-19 public health emergency, the SARS-CoV-2 experience is one from which lessons can be learned. Periodic monitoring of other pathogens responsible for nosocomial infections, using rapid methods like LAMP, would allow HCF CEOs to verify the quality of environmental hygiene protocols and implement improvements if necessary. This approach would facilitate the prevention of nosocomial infections and contribute to reducing the costs associated with managing these issues. In fact, while a rapid test with lower sensitivity may provide turnaround times of minutes or hours, standard tests can take days or even up to a week to deliver results for a suspected condition. In such cases, LAMP accessibility, defined by speed, ease of use, and specificity, can offer a reasonable trade-off for reduced SE, ACC, and PPV values, helping to break the chain of infection.

In contrast to RT-LAMP, RT-qPCR is known for its high sensitivity promoting the detection of low viral load. In clinical settings, this property facilitates the identification of positive patients with a low viral load [52]. However, in environmental settings, various external factors (e.g., hot temperatures, UV rays, detergents for surface hygiene, etc.) negatively affect the viral stability outside its host, leading to the degradation of nucleic acids, especially for an RNA virus like SARS-CoV-2 [51]. When applied to environmental settings, a highly sensitive technique like RT-qPCR could increase the probability of detecting non-viable pathogens. This, in turn, would impact the sanitation measures to use: sanitizing an environment based on a false positive result leads to a waste of materials which can also have an environmental impact (e.g., disinfectants can corrode surfaces and promote the release of surfaces’ components) and promote the increase in microbial resistance [53]. These findings suggest that RT-LAMP represents a good compromise compared to RT-qPCR considering that a clinical RT-qPCR kit was used to perform the comparison with RT-LAMP for the study of surfaces because no environmental RT-qPCR kit was available when the study began since most efforts were directed toward SARS-CoV-2 diagnostics [51,52,53].

The use of RT-LAMP to analyze environmental settings provides the best solution: having a high number of true-negative samples could reduce the probability of tracing false positives caused by the presence of nonviable microorganisms. This probability could be reduced by using solvents such as propidium monoazide (PMA) before the amplification. The advantage of PMA consists of penetrating into “dead” bacterial cells with a compromised membrane integrity, but not in living cells with intact cell membranes [54].

However, steps still remain to be taken to optimize the method. For example, the introduction of PMA into the amplification reaction to facilitate the exclusive recognition of live microorganisms. In addition, RT-LAMP could be used for wastewater or air monitoring for a deeper understanding of the transmission mechanisms of this and other pathogens. Performing rapid testing for the purpose of environmental surveillance encourages control interventions and rapid response. One more example is studying an outbreak in a community setting where it is necessary to evaluate the effectiveness of sanitation measures before reopening the facility.

Although the results obtained involve a small sample population, linked to the prevalent changes in the virus during the study and the use of more sensitive but laborious and expensive methods, such as digital PCR (dPCR), RT-LAMP constitutes a rapid, easy-to-use, and cost-effective method for the preliminary assessment of the hygienic condition of surfaces, to be confirmed in case of positivity by more sensitive molecular tests such as RT-qPCR or dPCR. This protocol could have application in the environmental surveillance of other pathogens of health concern and for evaluating the effectiveness of surface disinfection processes in high-risk environments such as HCFs and LTCFs frequently frequented by people with compromised immune defenses.

## 5. Conclusions

Although four years have passed since the SARS-CoV-2 pandemic began, few studies have been conducted on the environmental monitoring of the phenomenon. Most research, in fact, has focused on the clinical effects of the pandemic, which is the study of COVID-19. Monitoring SARS-CoV-2 in community settings by surface, wastewater, and air sampling provides a non-invasive way to monitor the pathogen’s circulation within populations. In this context, comparing different molecular detection techniques, such as RT-qPCR and RT-LAMP, is essential to optimize monitoring protocols and ensure accurate and reliable results.

Despite the low technique sensitivity, associated with the comparison with clinical RT-qPCR kit, our data confirm how RT-LAMP offers a valuable addition to the current testing strategies for SARS-CoV-2, including as a benefit for large-scale community screenings that are directly on-site, to assess the presence of viruses, and to check the status of sanitization protocols undertaken.

Understanding and implementing the limits previously discussed will make LAMP a valid alternative to other complex molecular strategies, to introduce in the routine surveillance not only for SARS-CoV-2, but also extending it to other pathogens implicated in nosocomial infections. Moreover, this preliminary study provides the foundation for the next steps in implementing the methods analyzed and writing comprehensive guidelines for the environmental monitoring of several pathogens.

## Figures and Tables

**Table 1:** Calculation of overall concordance (OC) among RT-qPCR and RT-LAMP through the percentage of positive and negative concordant samples.

RT-qPCR vs. RT-LAMP: Overall Concordance	
Positive concordant samples (True positives) No. (%)	8 (8%)
Negative concordant samples (True negatives) No. (%)	56 (56%)
OC No. (%)	64 (64%)

**Table 2:** Contingency table for the evaluation of Cohen’s Kappa (k) measure of agreement among the techniques (positive = +, negative = −).

	RT-qPCR vs. RT-LAMP: Cohen’s K Measurement			
		RT-LAMP		
		+	−	tot
RT-qPCR	+	8	17	25
	−	19	56	75
	tot	27	73	100
		Cohen’s K	0.0649	

**Table 3:** Comparison between RT-qPCR and RT-LAMP by studying Sensitivity (SE), Specificity (SP), Positive (PPV) and Negative Predictive Values (NPV), and Accuracy (ACC).

RT-qPCR vs. RT-LAMP	
SE (%)	32.0
95% CI	0.31–0.38
SP (%)	74.7
95% CI	0.74–0.76
PPV (%)	29.6
NPV (%)	76.7
ACC (%)	64.0

## Data Availability

The data presented in this study are available on request from the corresponding author due to privacy reasons.
